# The importance of spatial structure in biology

**DOI:** 10.1007/s13194-026-00760-x

**Published:** 2026-08-07

**Authors:** Margarida Hermida, James Ladyman

**Affiliations:** 1 School of Philosophy, Religion, and History of Science, University of Leeds, LS2 9JT Leeds, UK; 2 Department of Philosophy, University of Bristol, Cotham House, Bristol, BS6 6JL UK

**Keywords:** Biology, Explanation, Organicism, Physics, Reductionism, Spatial structure

## Abstract

Living things are highly complex and organised. This is often taken as an objection to reductionism and a point in favour of organicist views, which argue for the central role of the organism in all biological explanations. This paper argues that biological organisation often either is, or depends on, spatial structure, together with interactions among parts of the system understood in physical terms. We take spatial structure to include position, configuration, shape, directionality, and orientation of objects in space. Important features of the spatial structure of living systems include physical boundaries, such as membranes; cellular structure, which includes physical objects spatially arranged in certain ways; directionality and orientation with respect to internal and environmental signals; location, both of organisms in their environments and of components within the organism; and shape, including the shapes of organisms and their components at atomic, mesoscopic, and larger scales. The importance of spatial structure for explanations of the functioning of biological systems corroborates criticisms of genetic and molecular reductionism, but is compatible with physical reduction – the explanation of biological phenomena in physical terms – and part-whole reduction – the explanation of systems in terms of their parts, spatial structure, and interactions with each other and their environment.

## Introduction

Current biology is to a large extent a reductionist enterprise. Reductive approaches based on identifying the parts of an organism, characterising their physical structure, and studying their interactions, have produced explanations of complex biological phenomena, from physiology to behaviour and evolution. Some of the most successful advances in biology involve reductive explanations of fundamental biological processes such as respiration, the inheritance of characters, and the transmission of nerve impulses. The analysis of genetic sequences allows evolutionary biologists to infer evolutionary history and uncover hidden diversity in contemporary species. Explanations of the functioning of biological systems at various scales, from the metabolic activity of cells to the complex interactions that characterise ecosystems, ultimately rely on the physical transport of matter and the transference of energy, which connects electron chains in mitochondria to the transfer of chemical energy along with organic matter across trophic webs. It certainly seems as though much of biology is reductionist, both in the sense that it explains the properties of complex biological systems, such as organisms and ecosystems, on the basis of the properties of their parts and their interactions; and also in the sense that it provides physical explanations of biological processes. Where reductive explanations of biological phenomena are not yet available, great effort is put into discovering them (Weber, 2005, p. 20). The importance of physical explanation in biology is recognised in Love et al. (2017), Green (2018), and Batterman and Green (2021), among others.

However, reductionism is vehemently opposed by most philosophers of biology (Kaiser, 2011). Furthermore, currently there is a resurgence of *organicism*, a holistic philosophy that places the whole organism at the centre of all biological explanations (Mossio, 2024; Nicholson, 2014). Process philosophers, who argue that organisms are processes rather than things, consider their project to be continuous with the organicist tradition (Nicholson & Dupré, 2018, p. 11; Meincke, 2019), and several other philosophers of biology have recently endorsed organicist views that highlight the importance of *autonomy*, *organisation*, and *agency* in biological systems (Moreno & Mossio, 2015; Mossio, 2024; Reiss & Ruse, 2023).^1^ Nicholson (2014) cites three main reasons for the ‘return of the organism’ as a fundamental explanatory concept in biology:

(a) the realization that the Modern Synthesis does not provide a fully satisfactory understanding of evolution; (b) the growing awareness of the limits of reductionism in molecular biology; and (c) the renewed interest in the nature of life as a genuine scientific problem.

Notwithstanding the remarkable success of the Modern Synthesis in bringing together Darwin’s theory of evolution by natural selection with Mendelian genetics, it was formulated at a time when knowledge of molecular genetics was extremely limited (Hill, 2019). So it is not surprising it has limitations, but these are not limitations of reductive approaches per se. Concerning (b), as argued below, many of the criticisms of genetic and/or molecular reductionism are pertinent, but this is not the only kind of reduction, nor do its limitations give credence to organicism. Finally, concerning (c), the nature of life is one of the most exciting scientific problems, which was unduly eclipsed for a while by an excessive focus on genetics. However, it is doubtful that the best way to approach it is to take the organism as a fundamental concept. Indeed, arguably the problem cannot be tackled from an exclusively *biological* perspective because, assuming living things originated from non-living precursors, it is imperative to understand how living entities and processes relate to non-living entities and processes. In this context, reductive approaches that aim to explain the biological in terms of the physical hold more promise than holistic approaches that start from the organism itself as a required explanatory concept.

The move towards organicism, holism, and emergentism stems from the recognition that there is something missing from some traditional reductionist approaches. Biological systems are highly complex, hierarchically organised, and many of their capacities rely on the presence of heritable, evolutionarily acquired information (including, but not limited to, genetic information). Interactions between the parts of the system are very important, as are interactions with the environment - including other organisms and their parts. However, the complexity of living systems, the importance of interactions among their parts, and the importance of their environment, does not require strong emergence (Dupré, 2010, pp. 34-36), downward causation (Powell & Dupré, 2009, p. 60), or organicism (Nicholson & Gawne, 2015).

There is indeed something missing from some reductionist approaches, but part of what is required is more attention to *physical* aspects of organisms, their components, and their environments. In particular, spatial structure underpins much of the complexity of biological systems. The concept of *organisation* features prominently in anti-reductionist views but, although organicists take it to be an irreducible, fundamental biological concept that is both explanandum and explanans in biology (Mossio, 2024, p. 9), the organisation of biological systems can often be understood in terms of their spatial structure together with interactions among their parts. This spatial structure is partly directly inherited in cell division, and partly recreated in development through the use of genetic information coupled with environmental cues.

The importance of spatial structure is compatible with part-whole reduction and physical reduction. Part-whole reductive explanations are not restricted to consideration of the parts of a system in isolation, and the interactions among them, and their often nonlinear effects, are crucial. Contrary to organicist views, it is possible to explain the functioning of complex biological systems in terms of their parts, their spatial structure, and their interactions (including those with things located outside of the system). Both part-whole reduction and physical reduction are fruitful strategies in biological research that are independent of genetic reductionism. As Sarkar argues, “unlike genetic reductionism, physical reductionism has had a relatively successful career in biology”, and much of the philosophical confusion concerning reductionism in biology arises from “a conflation of genetic and physical reductionism” (Sarkar, 1998, p. 14). This paper defends only a version of the latter.^2^

The outline of the paper is as follows. Section 2 introduces different kinds of reductionism and identifies part-whole reduction and physical reduction as fruitful approaches to the search for explanations of biological phenomena. Sections 3 to 7 discuss different aspects of spatial structure that are important in biology. Section 3 focuses on physical boundaries, without which organisms cannot exist. Section 4 discusses the importance of cellular structure as an essential component of spatial structure in organisms, which also constitutes heritable structural information. Section 5 discusses directionality and how organisms can orient themselves in relation to internal and external cues. Section 6 argues for the importance of location, or relative spatial position, in biology, focusing on three aspects: the location of organisms in their environments, the relative positions of cells in multicellular development, and the location of structures and processes within cells. Section 7 discusses the importance of shape in relation to molecules and to organisms and their macroscopic parts. Section 8 argues that the spatial structure of organisms can be understood in physical terms, and that there is a kind of physical reductionist approach to understanding life that is a sensible and moderate position that accommodates the complexity of organisms and the processes that occur within them.

## Kinds of reductionism

There are many different notions of reduction and reductive explanation, both in general, and in the particular case of biology. Many critiques of reductionism focus on particular forms of reduction. Sometimes reductionism is equated with *unifactorialism*, i.e. the explanation of a complex phenomenon on the basis of only one causal factor. For example, Dupré (1993, p. 87) states that “[r]eductionism, in its broadest sense, is the commitment to any unifactorial explanation of a range of phenomena”, a procedure which is reductive insofar as it reduces the number of parameters in the explanatory model (Powell & Dupré, 2009, p. 55). This is also Van Regenmortel’s target when he argues that “[a] causal explanation is reductive in the sense that one factor is singled out for attention and is given undue explanatory weight on its own” (2002, p. 50). In most cases where scientists give causal explanations in terms of one single factor, however, they do not assume that no other factors are involved; rather, they are trying to identify the difference maker (Waters, 2007), relative to an implicitly defined set of background conditions. Evidently, simply ignoring relevant causal factors is inadequate, as even the staunchest reductionists recognise: “the temptation to simply ignore causal variables in explaining some outcome”, Rosenberg argues, “is clearly a mistaken strategy, and rightly criticized” (2006, p. 11).

Unifactorialism aside, there are four main kinds of reductionism in biology: (1) Microphysical reductionism; (2) Part-whole reductionism; (3) Physical reductionism; and (4) Genetic reductionism (and its sibling, molecular reductionism). Below it is argued that (2) and (3) are fruitful approaches to biological research, whereas whereas (1) and (4) face significant problems.

*Microphysical reductionism* is a kind of reduction that is somewhat popular among metaphysicians, but is never at issue in philosophy of biology, and for good reason. It is relatively rare that the entities and processes at the atomic scale or below are directly relevant to the explanation of biological phenomena, due to the typical scales at which biological phenomena occur. There are cases where processes happening at the atomic scale are relevant for the explanation of biological phenomena - for example, quantum tunnelling is important for the explanation of the functioning of respiratory chains in mitochondria, which in turn may have important implications for the explanation of many features of eukaryotes (Lane, 2011). However, most biological processes are not directly related to microphysics at all.

*Part-whole reduction* involves reducing an entity to its component parts and their interactions. This can be done at various levels or scales, and it may involve biological or non-biological parts. Examples of part-whole reduction include, for example, reducing a multicellular organism to its component cells and extracellular materials; reducing an ecosystem to individual organisms in individual-based ecological modelling; or reducing a cell to subcellular structures and molecules. In each case, it involves explaining the functioning of something larger on the basis of smaller component parts and their interactions. Dupré’s characterisation of reductionism as the claim that “the lynx is nothing but a collection of physical parts assembled in a certain way” (1993) is a statement of part-whole reductionism, as is Weber’s claim that “[r]eductionists (...) believe that once the parts of the system and their interactions are understood, there is nothing left for science to explain” (2005, p. 18). Taken literally, however, this statement is too strong, since there is also the rest of the world outside of the system, how it affects the system under study, and how it is affected by it in turn – all of which are appropriate things for science to explain (this is, of course, true of any physical system). Part-whole reduction, with or without taking into account the system’s interactions with its environment, can go a long way towards explaining the functioning of complex systems (Ladyman & Wiesner, 2020).^3^    

*Physical reduction* involves explaining the biological on the basis of the non-biological or physical (where ‘physical’ means relating to physical science, including chemistry, and is not restricted to microphysics). Although many physical reductions in biology involve relatively small molecules, this kind of reduction can apply to any scale at which biological processes take place. For example, the explanation of cell signalling in terms of changes in three-dimensional conformation of molecules is a reduction of a biological process to a physical one, and so is understanding the shape of a wing and the flight of a bird in terms of physical principles. This kind of reductionism is also a fruitful strategy in biology that can illuminate the workings of physical mechanisms that underlie many biological processes, and provide understanding of how life relates to laws and principles that also apply to non-living things.^4^

Our taxonomy of kinds of reduction differs from Sarkar’s (1998), who characterizes *strong reduction* as “the type of reduction where the properties of wholes are explained from those of the parts” and their interactions (Sarkar, 1998, p. 45), but adds that, when those parts are spatial parts, their interactions are physical interactions, and therefore equates strong reduction in relevant natural scientific contexts with physical reduction. Part-whole reduction often coincides with physical reduction, but not necessarily. For example, the physical reductive explanation of plant cell photosynthesis is also a part-whole reductive explanation since it involves splitting the plant cell into parts, namely, chloroplasts. However, physical reductive explanations in terms of entropy or free energy need not be part-whole reductive explanations, and the part-whole reduction of the behaviour of a social whole like a crowd or a market to the behaviour of individual agents is not a physical reductive explanation.

Finally, *genetic reductionism* is the view that explanations in biology should be given primarily in terms of genes or DNA molecules (a kind of reduction that may also be viewed as a particular type of unifactorialism). This is a view that has appealed to many, and was the basis of overblown claims concerning the significance of the human genome project. Notwithstanding the impressive achievements of molecular biology, genetic and/or molecular explanations are not the only kind of scientifically legitimate explanation in biology.^5^

Many anti-reductionist arguments in biology target genetic reductionism. For example, Dupré (2010, p. 28) criticises the view that “the genome contains *all* the information required to build an organism”. This is widely accepted to be excessively simplistic, as environmental signals can either activate or suppress genes. For example, temperature dependent sex determination in reptiles (Charnier, 1966) is a clear case where a (physical) environmental factor partly determines the phenotype through the simultaneous activation and suppression of the expression of genes associated with testis- and ovary-determining developmental cascades (Crews et al., 1994). Environmental factors are generally recognised to play important roles in development in less obvious ways as well, for example, a complete explanation of the development of vertebrate limbs should not ignore gravity (Mameli, 2005, p. 385). The fact that “genes themselves interact and are sensitive to triggers from the environment that can switch them on and off” (Reiss & Ruse, 2023, p. 2) is an important insight, but is only a problem for genetic, not physical, reductionism; likewise for the importance of “cellular, tissue, and organismal context in understanding the role of genes in development” (Nuño de la Rosa, 2018, p. 271).

Although not strictly equivalent to genetic reductionism, Rosenberg’s (2006, p. 12) *molecular reductionism*, which states that “for very biological fact, state, event, process, trend, or generalization”, there is a full and complete explanation which “will cite only the interaction of macromolecules”, suffers from the same problems, insofar as the restriction to the macromolecular scale is unwarranted. Even though macromolecules certainly play very important roles in many biological phenomena, there is no principled reason why all biological explanations should be given in terms of them, or even in terms of molecules more generally. As Laubichler and Wagner put it: “the fact that all biological objects are made of molecules does not imply that molecules are the most informative level of analysis” (2001, p. 61). Entities, processes, and events at various scales both above (e.g. whole cells; organs; organisms; groups of organisms) and below (e.g. quantum phenomena in respiration) can also play important explanatory roles in biology.

The recognition of the incompleteness of explanations given exclusively in molecular and/or genetic terms, however, does not require organicism. For example, from the fact that “local specification [of development] is not enough”, it does not follow that “the context of the organism as a whole is essential” (Nuño de la Rosa, 2018, p. 271) – it might be for some aspects of development and not others. The claim that “the properties of constituents cannot themselves be fully understood without a characterization of the larger system of which they are a part” (Dupré, 2010, p. 32) is not true. Many of the things that allegedly require explanation in terms of the whole determining the functioning of the parts, can be explained by the spatial structure and interactions of the physical parts of the system. For example, cells are spatially structured entities which cannot be treated as homogeneous assemblages of molecules, but their molecules and structures such as membranes and organelles have spatial structure.

Nicholson and Gawne highlight three main aspects of organicism: (1) a commitment to the centrality of the organism in biological explanation; (2) the importance of organisation as a theoretical principle, and (3) a defence of the autonomy of biology (Nicholson & Gawne, 2015, p. 361). In this paper, we argue that, on the contrary:

(1’) Which is the best level or scale for each particular biological explanation is an empirical matter that should not be decided a priori – some explanations might involve primarily interactions between molecules; other biological phenomena might be better explained in terms of supramolecular structures, cells, tissues, organs, organisms, ecosystems, or even bio-geochemical cycles; and yet others might need to appeal to electrons, photons, and quantum processes.

(2’) Organisation is indeed an important feature of many biological explanations, but it is not a proprietary theoretical biology principle; on the contrary, organisation is, first and foremost, spatial structure, and spatial structure (including its dynamical aspects and interactions among parts of the system) involves various physical properties of organisms and their parts. It is the fact that some purely molecular explanations neglect these physical aspects of organisms and their parts that renders them inadequate, because incomplete.

(3’) The autonomy of biology, which was an important issue in the twentieth century, is no longer at stake; it is evident that biology is its own discipline, with its own methods and theories. However, claims of the autonomy of biology can also be overstated – although biology is definitely a science in its own right and cannot be reduced to physics, physical explanations also play important roles in biology (Hermida & Ladyman, 2025, 5).

**Fig. 1:**
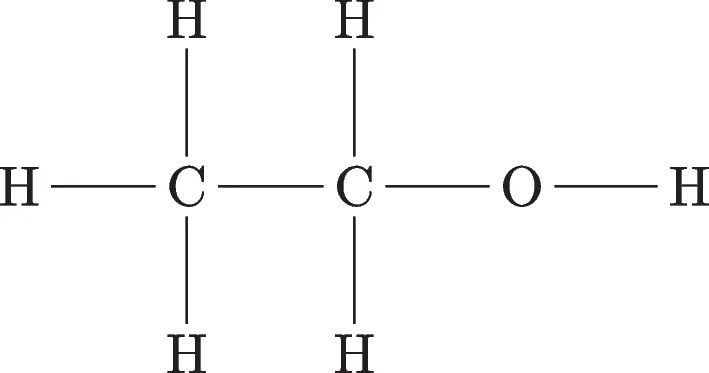
Ethanol

**Fig. 2:**
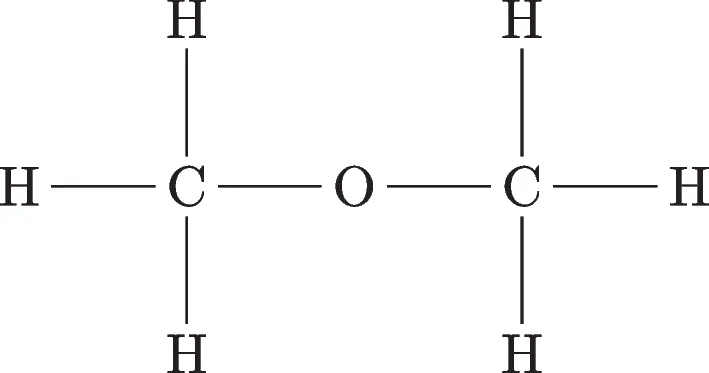
Dimethyl ether

Part of the reason why spatial structure is so important in biology is that it is so important in chemistry. There are substances that have exactly the same chemical composition, but different spatial structure, and so very different properties. For example, graphite, diamond, and buckyballs (buckminsterfullerene) are all composed exclusively of carbon atoms, but differ dramatically in their physical and chemical natures in virtue of their atoms being spatially arranged in different ways. Molecules with the same chemical composition can also have very different properties depending on the spatial arrangement of the atoms within the molecule; for example, both ethanol and dimethyl ether are composed of C_2_H_6_O  but have very different properties due to the different locations of the oxygen atom (Figs. 1 and 2).^6^

The next section considers spatial structure in biological systems in the form of *physical boundaries* that are essential for life.

## Boundaries

One of the most distinctive things about living entities is their activity; living things are highly active material objects - pieces of matter that, as Schrödinger (1944, p. 70) pointed out, “go on ‘doing something’, moving, exchanging material with [the] environment, and so forth”. Living organisms build, rebuild, and repair their own components, and keep themselves in a state of thermodynamic disequilibrium for far longer than a nonliving object typically would under similar circumstances. Living things achieve all this with *metabolism*, which involves the selective exchange of matter and energy with the environment. Through metabolic activity, living entities harvest free energy from the environment, which they use both to maintain and repair their own components, and to perform work (Schulze-Makuch & Irwin, 2018, p. 20).

If life requires metabolism, which is the controlled exchange of matter and energy with the environment, it follows that living things require a physical boundary that partially separates them from the environment (Hermida & Ladyman, 2022), in order to prevent the “bounded local environment” (Schulze-Makuch & Irwin, 2018, p. 19) interior to the organism from reaching equilibrium with its surrounding environment.^7^ It is essential that the boundary that separates the organism from its surrounding environment is semi-permeable to allow the metabolic exchanges of matter and energy. This is one of the most important features of cell membranes.^8^.

A putative exception to the rule that all living organisms require a boundary is the case of the alga *Bryopsis plumosa*.^9^ Many green algae are able to repair a partial rupture of the cell membrane by quickly sealing it with a protein and/or polyssacharide plug, in order to prevent loss of cell contents. Cells of *Bryopsis plumosa*, however, take this capacity to an extreme; extruded cell contents are apparently able to spontaneously regenerate an entire cell (Kim et al., 2001). When cells are cut and their contents spilled in seawater, cell organelles agglutinate into a circular mass which is temporarily without a cell membrane; within minutes, they become covered in a gelatinous envelope. This is not a lipid membrane; it is composed of polyssacharides, but it is also semi-permeable (Kim et al., 2001, p. 2012). A lipid cell membrane is then regenerated from the remains of the original cell membrane (Kim et al., 2001; Ram & Babbar, 2002).

Although this is a striking ability, it is not an exception to the rule that all living organisms require a physical boundary, nor does it amount to the “transient existence of life without a cell membrane” (Ram & Babbar, 2002). Firstly, the gelatinous envelope produced by the aggregated cell organelles has many of the features of a cell membrane, despite not being made of lipids. In particular, it is semi-permeable, allows for selective transport of materials, and holds the cell organelles together, acting as a “tentative defense wall against the external environment” (Ram & Babbar, 2002, p. 590). It works well enough as a (temporary) physical boundary, lasting for several hours, before a lipid membrane is regenerated.^10^ Secondly, the aggregates are never without membranes altogether; they include membrane-bound nuclei, mitochondria, chloroplasts, and internal cell membranes (Grossman, 2005, p. 2). The cell membrane is not produced *de novo*, but is regenerated from remnants of the original membrane (Kim et al., 2001). Thirdly, the Bryopsidales are coenocytic algae composed of multinucleate giant cells. The phenomenon described involves the aggregation of multiple cell nuclei (and other membrane-bound organelles), and therefore in some ways bears a stronger resemblance to the aggregation of independent cells in slime moulds (Bonner, 2009) and the re-aggregation of dissociated sponge cells (Lavrov & Kosevich, 2014) than it does to the regeneration of an ordinary eukaryotic cell.

Given how essential physical boundaries are for life, it is surprising to find several authors who seem to deny the existence of a boundary between the organism and the environment. For example, Turner (2009, p. 214) talks about the “essentially arbitrary boundary between organisms and the environment”; Griffiths and Gray (2001, p. 207) state that “there is no distinction between organism and environment”; and Smith (2017, p. 12) argues that “the embedded character of organisms (...) implies that there is no precise line of demarcation between organisms and environments”.

As it turns out none of these authors is denying the existence of a *physical boundary* between the organism and its environment; rather, they are all concerned with emphasizing how the interactions between the organism and its environment are important for biological explanations. Turner (2009) is developing Dawkins’s (1989) notion of the extended phenotype, i.e. the capacity of organisms to influence their environment (rather than merely being affected by it), for example by building structures such as nests; Griffiths and Gray (2001) are rejecting the notion that development involves the operation of genetic programs that operate independently of the environment; and Smith (2017) is highlighting the embeddedness of organisms in their environments. This latter point can be overstated. Organisms are indeed embedded in their environments to a certain extent, but they can, and do, move (or are transported) from one environment to a different one. The notion of adaptation requires the distinction between organism and environment, since organisms can be more or less adapted to the environments in which they find themselves. To be sure, the “lines of demarcation” (Smith, 2017, p. 12) may be imprecise, but they still demarcate.

Sensible reductionists do not deny the importance of the environment for organisms. As living things, organisms need to constantly undergo metabolic exchanges of matter and energy with the environment to maintain themselves, as Schrödinger (1944) pointed out. But, as noted above, metabolism requires semi-permeable boundaries. Anti-reductionists make much of the fact that organisms are open systems. Indeed, this is an important feature of organisms. More than that, they are steady-state systems, driven by the constant input of free energy, and matter, from the environment. But this does not preclude reductive explanations of such systems. Dupré argues that only closed biological systems (of which there are none) would supervene on their physical parts, whereas:

Bounded biological systems do not supervene on their physical parts because aspects of what they are depend on the context with which they interact, a context extending beyond any predetermined boundaries. (Dupré, 2010, p. 45)

It is certainly true that biological systems are open systems, but so too are many physical systems, for example, stars. If the criterion for something to be a closed system is that it has no interactions with the environment whatsoever, then there are no closed systems in physics either. What is meant by ’closed’ is that the system is ‘effectively’ closed in respect of some aspects of its dynamics (see Ladyman and Thébault, 2026). Living systems go to a lot of trouble to make aspects of their interior effectively closed systems. In physical terms, for a system to do any work it must have some internal dynamics. For example, for a living system to synthesize some substance, and to do it reliably and in a way that does not depend exclusively on the environment, it needs to separate some part of itself from the environment, and even from other internal systems. This separation is, of course, not complete. Biological systems are effectively closed for some aspects of their dynamics, even as they are open in others - for example, the circulatory system works particularly well when it is effectively closed off from the environment, and far less well when the boundary is breached; in contrast, the respiratory system cannot be closed off from the environment as gas exchanges need to happen continually.

It is not true, however, that the context which is relevant for biological systems “extends beyond any predetermined boundaries” (Dupré, 2010, p. 45). The only physical interaction that affects things at arbitrarily large distances is gravity and only the gravity of the Earth and the Moon are relevant to life on Earth as gravitation is very weak compared to electromagnetism and mechanical forces which act locally (chemical interactions require spatial proximity because they are based on electromagnetism). The need to take into account environmental interactions neither implies that the organism has no boundary that separates it from the environment, nor that environmental effects are anything but local.^11^

## Cellular structure

Cells are highly structured objects. The spatial structure of cells, or cellular ‘architecture’, “is what ultimately distinguishes a living cell from a soup of the chemicals of which it is composed” (Harold, 2005, p. 544). Even bacterial and archaeal cells, which lack the structural complexity of eukaryotes, are not homogeneous bags of chemicals. The circular bacterial chromosome is located in the centre of the cell, forming the nucleoid, “a discrete, well-defined physical object” (Kleckner et al., 2014), and ribosomes are mostly distributed at the periphery of this structure. In addition, biophysical and biochemical interactions among inhomogeneous macromolecules in the highly crowded interior of the cell result in the production of clusters or multiplexes of macromolecules that alternate with relatively uncrowded ‘reservoirs’ of cytosol, through which molecules can easily diffuse (Spitzer, 2011). Spatial separation of different processes allows them to proceed concurrently and without unwanted interactions. For example, there is a significant degree of spatial segregation between the transcription and replication machineries within bacterial cells, which allows for both to operate simultaneously and thus achieve higher growth rates (Jin et al., 2017).

The spatial segregation of different components and different processes within the cell is particularly noticeable in eukaryotic cells. Having resulted from the endosymbiotic fusion of an archaeon and a bacterium (which evolved into the mitochondria), eukaryotic cells are more than a hundred times larger, and far more complex, than either of those. Besides the mitochondria and, in plant cells, chloroplasts and other plastids, they include a wide array of membrane-bound organelles, a sophisticated cytoskeleton, and an intracellular transport system. Different processes and different steps of the same process often take place in regions of the cell that are topologically separated by membranes. For example, DNA transcription takes place in the nucleus; RNA is then transported out of the nucleus and protein synthesis takes place in ribosomes in the cytoplasm; ATP production takes place in the mitochondria; etc.

Many of these structures cannot be produced *de novo* by cells, and have to be inherited in bulk from previously existing cells. This is notably the case for the cell membrane, which can only be extended, but not built *de novo*, as phospholipids only insert themselves into existing membranes (Harold, 2001, p. 105). Furthermore, membrane proteins can only be integrated into “translocation competent membranes”; but since the translocators themselves consist of integrated membrane proteins that require translocation into the membrane, it follows that cell membranes always have to originate from previous membranes (Blobel, 1980, p. 1498).

This means that the membranes of contemporary cells are *physically continuous* across reproductive events throughout the entire history of life on Earth, since the earliest cells (Fields & Levin, 2018). Upon cell division, each cell receives roughly half of the membrane and half of complex organelles such as mitochondria and plastids, which need to be transmitted intact and in a functioning state to the daughter cells. Some internal membranes, such as the nuclear envelope and the Golgi complex, are disassembled into small vesicles and tubes during cell division and then reassembled in the daughter cells (Matlin, 2022, pp. 239-240). Other structures are built *de novo* by the cell from genetic instructions. However, in many cases their spatial organisation still needs to be inherited from previous cells:

[T]opogenetic sequences on transported proteins are deciphered by prepositioned cellular machinery to target proteins to their correct destinations. When components of this sorting machinery are synthesized, they depend on preexisting copies of themselves to make sure they end up in their correct locations. (Matlin, 2022, p. 238)

There is also a chicken-and-egg situation concerning the cell’s transcription and translation machinery. Information for producing ribosomal subunits is coded in the DNA, and the cell can produce and assemble these *de novo*. Nonetheless, unless *at least some* ribosomes are transmitted in bulk in cell division, the cell cannot read its own instructions for how to build ribosomal subunits in the first place.^12^

Dupré (2010) is right to point out that the genome is not the only thing that is transmitted in reproduction – although even genetic reductionists would have to agree that cells need to inherit, at the very least, the transcription and translation machinery that allows them to read the genes in the first place – much more than this is transmitted. As Griesemer (2000) argues, reproduction always involves the material transfer of organised *physical parts* of parents to offspring. In addition to genetic information and some epigenetic factors, cells inherit functional material components in certain spatial configurations. These spatial configurations contain *structural* information (Morowitz, 1968) that is transmitted to the daughter cells upon reproduction. In the case of DNA, it is the specific sequence of base pairs that contains information.^13^ In the case of cellular structure, structural information is present in the transmitted structures themselves, i.e. the specific complex physical objects such as membranes, and spatial and topological relations between these, such as, for example, the location of specific molecules *within* a membrane-bound organelle, and the location of certain proteins in the cell membrane, which provides positional information that allows the cell to target proteins to specific locations (Matlin, 2022).

Both the functioning of the cell, and the inheritance of many complex features of cells, involve the spatial and topological structure of the cell and its components as a necessary explanans, which rules out strictly molecular accounts that disregard spatial structure. As Dupré notes,

One cannot infer the behaviour of a cell by treating it as a bag of chemicals; one might begin to make progress by describing the intermediate structures viz., ribosomes, Golgi apparatuses, and so on and the heterogeneous distribution of various molecules in relation to such things. (Dupré, 2010, p. 141)

Indeed, one cannot infer, or explain, the behaviour of a cell by treating it as a homogeneous bag of chemicals – just like one cannot infer, or explain, the behaviour of a molecule by treating it as a bag of atoms, while disregarding its spatial structure. The cell is a spatially structured assemblage of molecules and other physical objects, and some of its behaviour can be inferred, and explained, by taking into account the parts (not exclusively molecular), their interactions, and their spatial relations. Part of this spatial structure is directly generated by the biophysical and biochemical interactions among molecules in a crowded environment (Spitzer, 2011); part of it involves the dynamical self-organisation of cellular structures such as microtubules and cytoskeletal components (Nicholson, 2019); part of it is inherited in bulk from previous cells; and some is rebuilt by the cell from DNA sequences also inherited from previous cells, with the help of positional information provided by the placement of certain molecules. Since at least some of the spatial structure of cells is “propagated by mechanisms that are, at least in part, independent of molecular chemistry” (Harold, 2005, p. 560), purely molecular reductionism is untenable. Nonetheless, all this highly complex spatial structure can be understood in physical terms - it is composed of *physical objects*, such as membranes, ribosomes, molecules, etc, and their spatial relations, i.e. their relative positions in space. As Nicholson (2019) points out in his forceful defense of an anti-reductionist view of the cell, our growing understanding of cellular complexity and the dynamical, fluid, and stochastic nature of the functioning of many cellular processes requires, among other things, that we

“seriously consider how the ideas of non-equilibrium thermodynamics and complexity theory, and even those of condensed matter physics and quantum mechanics, may be brought to bear on the interpretation and explanation of the phenomena we investigate” (Nicholson, 2019, p. 124).

Nicholson is certainly right in emphasising the stochastic, dynamic, and non-mechanical nature of many of the cell’s components and processes, but dynamical physical structures are still physical structures, and their physical properties are important for understanding their functioning. For example, microtubules are “constantly polymerizing and depolymerizing” (Nicholson, 2019, p. 111), but in order to understand this process, it’s also important to know that microtubule polymerisation and depolymerisation are regulated by physical properties of the cytoplasm, in particular its viscosity (Molines et al., 2022). Although this paper focuses primarily on spatial structure, other physical aspects of cells also play important roles in complementing molecular descriptions of the cell.

That cellular spatial structure is important for understanding cell biology does not mean, however, that all explanations of cellular processes must take the cell as a whole to be the privileged level of explanation. The whole cell is rather the *starting point* of the investigation. According to Matlin (2022), the epistemic strategy of cell biology involves localisation and decomposition, two crucial strategies often employed in the study of complex systems (Bechtel & Richardson, 1993). More specifically, cell biology studies might involve, first, a morphological description of cells and their parts; then, the localisation of the phenomenon under study in a particular structure within the cell, and the chemical analysis of cell parts obtained by cell fractionation (literally breaking the cells apart and centrifuging the contents); finally, experiments involving specific cell parts are carried out to test hypotheses regarding the mechanism responsible for the phenomenon (Matlin, 2022, pp. 270–275).

Often, the cell structures, the identification of the specific molecules involved, and their interactions, are best studied *in vitro* or in ‘cell-free systems’ that retain little of the spatial organisation of living cells (Matlin, 2022; Wagner et al., 2023). This does not mean that researchers using these strategies assume that the cell is a perfectly aggregative system (Bechtel & Richardson, 1993, p. 25), or that they are necessarily ignoring interactions. What it means is that reductive methodologies that decompose the system into parts are well suited to tackling complex biological phenomena in a piecemeal fashion. This is no less the case when the phenomenon under study is itself an interaction. For example, the study of the interactions between the nucleus and the mitochondria has been successfully investigated using a pure *in vitro* approach (Gaspari et al., 2004). These reductive approaches have several advantages: they facilitate more fine-grained manipulability of the target system; they allow researchers to exclude unwanted interactions with other cell components in order to zoom in on a particular set of interactions; and they also allow for the study of particular combinations of cell components that are not viable and for that reason cannot be studied *in vivo*, but which are highly informative as to how and why certain mechanisms fail.

Anti-reductionists are quick to conclude, from the fact that not everything can be reduced to molecules, that every cellular process must be understood in the context of the whole cell, seen as an undecomposable whole. However, as Wagner and Laubichler say, “[f]inding the appropriate level of description is ultimately an empirical question. Privileging any particular level of description *a priori* is bad metaphysics and even worse science” (2000, p. 27); this applies to higher levels as well – no particular level should be privileged *a priori*. Importantly, the ‘higher level objects’ are not limited to cells and organisms. There is a lot going on between the atomic scale and scale of the whole cell and, often, the structures that are most relevant for the explanation of subcellular processes are intermediate structures, such as organelles and membranes. For example, the chemiosmotic process of respiration discovered by Mitchell makes ineliminable reference to “supramolecular features” (Mitchell, 1961, p. 145), in particular, the anisotropic charge-impermeable mitochondrial membrane on which the respiratory enzymes are located, and the spatial arrangement of the electron transfer chain on this membrane. The process of chemiosmotic coupling “demands a topologically closed system such as a vesicle” (Harold, 2001, p. 86), without which it cannot function. Even in bacteria, hyperstructures such as macromolecular ’factories’ and enzyme assemblies contribute to the spatial structure of the cell, and may be crucial for explaining processes such as the cell cycle (Norris et al., 2007, 1999).

Explanations involving these supramolecular features do not necessarily involve the context of the whole cell. Matlin argues that the “ontological primacy of the living cell” is preserved in membrane-bound organelles, or even vesicular fragments of these organelles, as they “retain sufficient *cellness* in isolation to permit functional explanations of biological phenomena to be achieved within the context of their ‘*milieu*”’ (2016, p. 86). But what this ‘cellness’ consists in is a particular spatial structure, namely a continuous membrane featuring asymmetrically distributed proteins (Matlin, 2016). And this spatial and topological structure can be understood in physical terms – as when Mitchell provided a physical explanation of how oxidative phosphorylation is coupled to electron and proton transfer by a transmembrane chemiosmotic mechanism (Mitchell, 1961).

## Directionality

Many, if not most, biochemical reactions within the cell are vectorial, i.e. they have an intrinsic direction in space, often due to their association with membranes (Harold, 2001, p. 111). The chemiosmotic process involved in respiration, discussed in the previous section, is a prime example of a directional process, but many metabolic reactions are also directional. The fact that proteins and other molecules have shapes, “come with clefts, cavities and sometimes channels, (...) and possess intrinsic asymmetry” (Harold, 1991, p. 348) has important consequences for the directionality of chemical reactions within the cell. Reactions catalysed by enzymes proceed along a particular spatial trajectory within the protein, as recognised by Mitchell and Moyle (1958); DNA elongation proceeds along one particular direction, and while the 3’-5’ strand can be copied directly, the 5’-3’ strand must be copied in short segments; ribosomes read mRNA in one particular direction and polymerise proteins in one particular direction; and so on (Harold, 1991).

These spatially oriented vectorial processes at nanometer scales are surely the basis for the generation of spatial structure at the scale of cells and organisms, but there are further mechanisms that allow organisms to bridge these scales and generate what Harold (1991) terms ‘vectorial physiology’. For example, in Fungi, the driving force for apical growth is the hydrostatic pressure which is exerted uniformly on the cell wall by the cytoplasm; but the tip of the hypha yields to it locally – an example of the more general phenomenon of localised compliance with global force – by stretching and secreting new wall, a process which is carried out by enzymes carried in vesicles that are transported to the tip and exocytosed there (Harold, 1991, pp. 366-367).

Directionality is particularly important for multicellular development and growth. The problem of development in multicellular organisms is, as pointed out by Laubichler and Wagner (2001, p. 57), “basically one of spatial differentiation and growth”, and they highlight the spatial dimension of development and the context-dependency of biochemical processes as particularly problematic for (molecular) reductionism. Some of the features of development that Laubichler and Wagner (2001) argue are incompatible with reductionism are actually not that problematic even for molecular reductionists. For example, the fact that one molecule can perform multiple functions or that the same function can be performed by more than one molecule are perfectly compatible with token or mereological reductionism (Delehanty, 2005) and, often, context can itself be reduced to a molecular explanation. For example, the fact that the same gene plays different roles in different organisms is not a case of “the same biochemical pathways (...) leading to different morphological outcomes” (Laubichler & Wagner, 2001, p. 63), because the complete molecular pathway in the two organisms is different, due to the presence of different genes, different proteins, and so on (Frost-Arnold, 2004, p. 82).

Frost-Arnold (2004) argues that the context objection can nonetheless be maintained if there are cases where spatial organisation cannot be reduced to the molecular level. Examples may be hard to come by since, in many cases, the way that spatial organisation makes a difference to development is precisely due to differences in molecular distribution both within and across cells and tissues. For example, in many animal species, early embryonic development is determined by oocyte polarity, which consists in the asymmetric distribution of molecules and organelles within the cell. However, there are also cases where an environmental signal determines embryonic development. Frost-Arnold discusses two hypothetical cases where the direction that determines development is not established through the asymmetric distribution of molecules or other structures within the cell:

Suppose we have a fertilized egg which is, from a molecular point of view, spherically symmetric (...). Here is the crucial step: now suppose the half ’on the bottom of the organism’ always becomes X while the half ‘on the top of the organism’ always becomes Y – where which half is the ‘bottom’ is determined by the orientation of the embryo with respect to gravity, or perhaps with respect to the mother’s uterus in certain animals. We would then have, *ex hypothesi*, two situations that are molecularly identical (the two halves of the embryo), but developmentally different. (Frost-Arnold, 2004, pp. 84–85)

In cases like these, the cell may be symmetric in terms of molecular distribution, but there is an environmental factor that determines the axis of the embryo. In one case, it is gravity – a physical force that can used by biological systems to determine their orientation in space. In the second case, it is direction with respect to the maternal organism, which must also be detected by the cell in some way – for example, through asymmetric chemical signals or mechanical forces. It might be worth looking at an actual example of how environmental cues can provide directional information in development:

The zygotes of the brown algae *Fucus* and *Silvetia* spp. (...) are, to judge by their appearance and behavior, spherically symmetrical. The axis of the developing embryo is established when the zygote germinates, at a locus determined by the direction of incident light. Zygotes will germinate even in the dark, at a locus that corresponds to the site of sperm entry. When all the data are in, will these early stages of morphogenesis be wholly comprehensible in terms of the chemistry of the participating molecules? Obviously not; but they should be comprehensible as a spatially organized system of molecules that can take directions from environmental vectors. (Harold, 2005, p. 560)

Spatial structure is a problem for molecular reductionism, since a complete explanation of these processes often involves non-molecular factors, for instance gravity or light. But they can be physically explained in terms of environmental vectors, whether these are molecular gradients, or physical forces such as gravity, mechanical forces (e.g. mechanical tension from adjacent cells, hydrostatic pressure from a fluid-filled cavity in the gastrula), or electromagnetic radiation (light). All these factors can be understood in physical terms, and they bring about molecular changes, including differences in gene expression, in different parts of the embryo.

Another interesting example of directionality is the case of directional growth in plants. Plants are able to detect several environmental cues, including light, temperature, and gravity, using these as information for directional growth. Gravity sensing, for example, occurs both in roots and shoots: roots grow downwards in the direction of gravity, in a process of positive gravitropism, whereas primary shoot stems grow upwards in the opposite direction to the gravity vector (negative gravitropism). Gravitropism involves, first, the detection of the direction of gravity, which takes place in specialised gravity-sensing plant cells, the statocytes; then, the conversion of this information into biochemical signals, and the transmission of these signals to other cells and tissues through biochemical signalling, involving the asymmetric distribution of the plant hormone auxin, which then leads to asymmetric organ growth via differential cell division and/or elongation (Kawamoto & Morita, 2022). Statocytes contain statoliths, which are amyloplasts filled with starch-rich granules that sediment at the bottom of the cell. The localisation of the amyloplasts in the bottom region of the cell then generates a biochemical signal through the translocation of a protein, LZY4, from the amyloplasts to the nearby plasma membrane, generating cell polarity (Nishimura et al., 2023). This in turn recruits another protein, RDL, which in turn regulates asymmetric distribution of auxin in plant tissues, resulting in directional growth (Furutani et al., 2020).

The explanation of gravitropism in plants involves: (1) a gravity sensing process that is explained in physical terms, based on the extremely simple physical principle that dense granules that are heavier than the surrounding cytoplasm tend to fall to the bottom of the cell (Kawamoto & Morita, 2022, p. 1641); and (2) the coupling of this physical process with a biochemical signalling pathway involving proteins and the plant growth hormone auxin, whose asymmetric distribution in plant tissues determines directional growth through regulation of gene expression and other mechanisms. As in the case of embryonic development, the orientation of the growing tissue (in this case, relative to the gravity vector) is detected in subcellular structures through a biophysical mechanism, and is then conveyed to other cells and tissues through biochemical signalling. No reference to the whole plant is required, and each step involves only local physical and chemical processes, which together and through their interactions bring about the complex biological phenomenon of directional plant growth.

## Location, location, location

The location, or relative position of an object in space with relation to other objects, is an important physical property that partly determines how a system will evolve over time, what interactions the object can engage in, etc. The location of organisms in suitable environments is a prime determinant of biological outcomes, but equally important for the explanation of biological functioning are the relative positions of objects, such as cells and subcellular components, within organisms. The locations of organisms in their environments, and the relative spatial positions of components within the organism, account for much of the context dependency of biological functioning.

### Location of organisms in their environments

Organisms are dynamically stable and relatively robust physical objects (Hermida & Ladyman, 2022) within environments that are habitable for them, but often die when placed in very different environments from the ones to which they are adapted. Most of the universe is extremely hostile to life. The location of an organism within its specific habitat is important to its survival and reproduction. Territories within a habitat can vary dramatically in quality and resources, and many ecological trade-offs that organisms must engage in have to do with the costs and benefits of acquiring and maintaining good territories. For example, migratory birds benefit from arriving early at their summer locations so they have the pick of the best territories, which contain more resources and are able to support larger and healthier broods, but not too early that they might experience unfavourable weather conditions which can decrease survival. Organisms also engage in homeostatic regulation by choosing different locations within a habitat at different times. For example, ectothermic organisms such as fishes and lizards engage in behavioural temperature regulation by moving to warmer areas to increase body temperature after foraging in colder spots.

The importance of environmental interactions means that anti-reductionists have a point when they say that the behaviour of a biological system is not *fully* determined by the behaviour of its parts, since environmental factors also partly determine its behaviour. For example, the behaviour of the Moon cannot be fully explained by the behaviour of its parts, because it is partly explained by its interaction with the Earth, but this is a gravitational interaction which can be adequately explained in physical terms. Nonetheless, even if part-whole explanations are always incomplete in this sense, they still explain a great deal about the system under consideration. We take it that anti-reductionists would not object to an explanation of the functioning of a TV set in terms of the behaviour of its parts, although a full explanation must also include the electrical input from outside the system and the dissipation of heat to the surrounding environment. Environmental interactions do not pose a problem for physical reduction, since there is no reason why interactions with things located *outside* of the object of interest should not be part of the explanation, as long as they bring about their effects by some physical means.

### Location of cells in embryonic development

The development of multicellular organisms is a notoriously complex process involving growth, cell division, and cell differentiation, which robustly results in the generation of complex morphological features. Laubichler and Wagner criticise the inability of molecular reductionism (Rosenberg, 1997) to account for the fact that developing organisms “have a three-dimensional structure and are context-sensitive in space and time” (Laubichler & Wagner, 2001, p. 58). Because molecular reductionist explanations fail to take into account the three-dimensional structure of the organism, and how developmental processes depend on spatial and temporal context, they cannot – even with complete knowledge of all molecular interactions – “explain how these molecular interactions are causally linked to the spatial and temporal differentiation of particular morphological structures” (Laubichler & Wagner, 2001, p. 63).

Indeed, for morphological structures to be adequately produced in development, it is essential that different parts of the embryo develop in different, and coordinated, ways. Thus, spatial structure is important for explanations of development, and strictly genetic and/or molecular approaches that fail to include this aspect face serious limitations.

For example, canalization of gene expression and determination of developmental pathways in the *Drosophila* blastoderm is explained on the basis of how positional information in the early embryo, which is made available to cells through the differential expression of gap genes in broad domains (anterior, posterior, and terminal), under the control of maternal cues already present in the egg, generates different basins of attraction, within which different attractors canalize cell differentiation. In effect, these basins of attraction determine which attractors are operational for each cell, as a function of its position on the anterior-posterior axis (Manu et al., 2009).

Lineage specification in mammalian embryonic development also partly depends on the location of cells in the blastula. The specification of the first cell lineages in mammalian embryogenesis involves “cell-cell and cell-environment interactions, geometrical constraints, changing physical forces and adaptive gene regulatory networks” which together “drive the formation of the first mammalian embryonic lineages and their robust organisation into a three-dimensional structure” (Płusa & Piliszek, 2020). The *trophectoderm* (TE) is the first cell lineage to be specified from cells that are located on the outside in the morula (i.e. the embryo prior to the formation of the blastocyst). These cells will go on contribute to the formation of the placenta. The cells on the inside form the inner cell mass (ICM), which contributes to the formation of all foetal tissues.

The fact that the location of the cells partly determines cell lineages need not, however, motivate the conclusion that cell fate determination can only be understood in the context of the whole embryo, or that it cannot admit of reductive explanation, and in particular physical reduction, i.e. explanation in terms of physical processes. Cell fate determination depends on *relative spatial position* and interactions between cells, and can be further investigated at the cellular and subcellular scale. In fact, physical processes can explain how cells come to be located in the particular layers where they are located in the first place. For example, the direction of cell division in the morula results in one of two outcomes: either one of the daughter cells is located on the inside and the other on the outside, or both are located on the outside layer. Among the cells located on the outside layer, however, there is heterogeneity in mechanical tension, caused by differences in contractility which in turn result from asymmetric inheritance of the apical domain in cell division. Above a specific threshold of mechanical tension, a surface cell becomes internalised, and will then form part of the ICM (Maître et al., 2016).

Furthermore, the way in which the relative position of cells within the embryo brings about the contextual differences that Laubichler and Wagner (2001) are concerned with can be understood in terms of physical processes. In many cases, as Delehanty points out, “[b]ecause the activities engaged in by molecular entities do not act at a distance, spatial and temporal aspects of biological context can be explained in molecular terms” (Delehanty, 2005, p. 733). In other cases, the explanation involves, in the first instance, the action of *physical forces*, such as gravity and electromagnetism. How these forces act depends on spatial structure – including spatial separation and the relative spatial positions of the components of biological systems – is indeed important for at least some developmental explanations. Again, though, this is only problematic for molecular, but not physical reductionism, which involves the explanation of biological phenomenon in terms of any physical processes. For example, in developing embryos (both in animals and plants), mechanical forces such as tension and compression can function as *positional information* which is then translated into biochemical responses that can affect gene expression which determines the behaviour and fate of cell lineages (Hernández-Hernández et al., 2014). Biochemical changes in the cell can in turn also result in changes to the mechanical properties of cells (such as rigidity, adhesiveness, and so on), such that positional information results from the complex interaction and feedback among these physical and biochemical processes (Hernández-Hernández et al., 2014).

### Location of structures and processes within the cell

As discussed in section 4, cells are spatially structured, and most processes that take place within a cell are *localised*, i.e., they occur in discrete, identifiable locations (Bourke et al., 2023). Thus, the transcription of DNA into RNA occurs in the nucleus, and then the mRNA exits to the cytoplasm and is translated in the ribosomes. However, translation does not take place evenly across the cytoplasm, but tends to be localised to where particular proteins are needed. There are several advantages to local translation occurring where protein is needed. On the one hand, it’s energetically less costly to transport one mRNA molecule which can then be used as a template for many protein molecules; it also minimises unwanted cellular interactions that might happen if proteins were transported across the cell; and it enhances the cell’s ability to quickly and effectively modulate its proteome (Bourke et al., 2023).

An extreme case of localisation of protein translation occurs in neurons. Neurons are highly polarised and spatially structured cells, with axons that can extend for more than 1 metre in humans (Biever et al., 2019). Synaptic plasticity, which is required for cognitive processes such as learning, memory, and behaviour, involves long-term changes in synaptic strength, which are brought about through altered protein composition at synaptic sites. These changes are mainly achieved through local protein synthesis taking place near the synapse, which is not only quicker and more energetically efficient than transporting proteins across the cell, but, crucially, also allows for greater synaptic specificity. Localised protein synthesis allows for the specific potentiation or depression of a synapse in response to stimuli, while nearby synapses are not affected (Rajgor et al., 2021). Local protein synthesis near synapses is supported by the localisation of mRNAs, which are produced in the nucleus, to the required locations. RNAs self-assemble by liquid-liquid phase separation, forming granules that are then actively transported across the length of axon by motor proteins along cytoskeleton tracks (Dalla Costa et al., 2021). Once mRNAs they reach their destination, translation and protein synthesis is modulated by an array of regulatory RNAs, also localised to this region (Biever et al., 2019).

The need for local control over certain processes in specific locations within the cell is also involved in the explanation of an intriguing feature of eukaryotic cells - the persistence of DNA in bioenergetic organelles, namely mitochondria and chloroplasts. Over the course of evolution, most mitochondrial and plastid genes have been transferred to the nucleus. There are good evolutionary reasons for that, including better efficiency and economy of resource use, decreased susceptibility to mutation caused by free radical by-products, and the evolutionary advantages of recombination for purging harmful mutations (Allen, 2003). However, a subset of mitochondrial genes (and plastid genes in plants) has been retained in these organelles. The genes that have *not* been transferred to the nucleus code for essential components of the electron transport chain (Hill, 2019), and the reason they have been retained there is that these genes need to be physically located close to, and in the same cellular compartment as, their products. This co-location facilitates direct regulatory coupling, a hypothesis termed ‘co-location for redox regulation of gene expression’, or CORR (Allen, 2003), for which there is ample empirical evidence and theoretical support (Hill, 2019; Lane, 2011). Maintaining membrane potential and an efficient flow of electrons across the electron transport chain requires local control by redox-sensitive regulatory products, which is achieved by having “the right genes in the right place” (Lane, 2011, p. 860). The mitochondria are thus able to quickly respond to changes in free radical leak resulting from any imbalance in the delicate biophysical mechanism of electron transport (Lane, 2011). Since transcriptional regulation takes place locally, mitochondria can adjust their performance quickly and “without a requirement for prior consultation and coordination with what may be regarded as a nucleo-cytoplasmic bureaucracy” (Allen, 2003, p. 31).

Location in space relative to other objects is an important aspect of spatial structure, and the above examples demonstrate how location is important to the explanation of many biological phenomena. The importance of location is a natural consequence of the fact that biological processes always involve physical interactions. Things act on other things and bring about their effects either through chemical interactions (which require proximity) or through local mechanical forces.

## Shape

Shape is an object’s configuration in space which results from the relative positions of its parts. It captures “all the geometrical information that remains when location, scale and rotational effects are filtered out from an object” (Dryden and Mardia, 2016, p. 1, based on Kendall’s 1984 definition). Shape is involved in many biological phenomena, as organicists observe (von Bertallanfy, 1952, p. 158). Shape is important for different kinds of biological processes. The next three subsections consider molecular shape, the shapes of cells, and the macroscopic shapes of organisms and their parts respectively.

### Molecular shape

One of the most striking chemical features of living systems is their chirality. All life on Earth uses exclusively L-chiral amino acids in proteins and R-chiral sugars in nucleic acids, while abiotic sources of these compounds tend to be racemic mixtures of both enantiomers. Chirality is particularly important for biopolymers, such as DNA and proteins, as homochiral polymers are considerably more stable, and polymerisation of nucleic acids is much more efficient if the monomers have the same chirality as the template, and each other (Bonner, 1995).

More generally, the three-dimensional shapes of molecules determine many of their physical and chemical properties. This is particularly noticeable in the case of proteins, which can assume extremely diverse shapes and perform various roles in biological systems. Hydrophobic and hydrophilic interactions, electromagnetic attraction and repulsion, hydrogen bonds, and van der Waals forces make proteins bend and fold, firstly into simple patterns like *α *-helices and *β *-sheets, and then into more complex three-dimensional conformations that allow proteins to fulfil various functions. In fact, “[t]he explanation for the functional versatility of proteins is not chemical so much as physical” (Harold, 2001, p. 37) – it is the shapes that proteins fold into, as well as their dynamical nature and the specific ways in which they change shape when interacting with ligands, that allow them to perform various roles in biological systems, from structural fibres and cytoskeleton components to enzymes and molecular motors. Enzymatic activity, for example, depends on the shape of the enzyme, which partly determines which substrates it can bind to, and then the shapes of the two molecules, together with thermodynamic constraints, determine the conformational changes the enzyme undergoes during binding.^14^

The shape of molecules is also crucially involved in self-assembly processes: firstly, because it affects whether and how molecules spontaneously bind to each other and form aggregates; secondly, the shape of the component molecules determines how they arrange themselves in space and sets the curvature of the aggregate, determining its shape (Hyde et al., 1996, p. 141), which in turn will affect how it behaves and interacts with its surroundings. Chirality adds another layer of complexity to these effects, by setting a preferred *tilt* to the arrangement and favouring particular forms of close-packing (Hyde et al., 1996, p. 141).

Molecular shape is particularly complex in the case of the extremely long and highly compacted DNA molecules. Because DNA molecules are so large, they form complex structures at different scales, from the double helix conformation of the two DNA strands, to its tight wrapping around histones, and then more complex three-dimensional structures, up to the shape of entire chromosomes. Furthermore, all this structure is dynamical and can undergo quite significant changes, especially during cell division. Since chromatin architecture spans a range of scales, one important question is “how activity of molecules at the nanometer scale can lead to complex and orchestrated spatial organization at the scale of chromosomes and the whole nucleus” (Mirny & Dekker, 2022). This seems to involve at least three distinct mechanisms: (1) tethering of specific loci to nuclear landmarks; (2) spatial compartmentalisation of chromatin, driven by molecular affinities, resulting in spatial segregation of active and inactive regions of DNA; and (3) active loop extrusion of certain portions of DNA, which is carried out by motor. Chromatin architecture results from the operation and interaction among these mechanisms, as well as from topological constraints on chromosome folding that result from biophysical properties of the molecule (Mirny & Dekker, 2022).

The complex three-dimensional folding of chromosomes determines which DNA sequences end up being spatially located close to which other sequences, and are therefore more likely to interact. This feature is highly relevant for transcriptional control, especially in eukaryotic genomes, where regulatory elements that can either activate or silence gene expression can be positioned thousands or millions of base pairs away from the genes they regulate (Hafner & Boettiger, 2023).

### The shapes of cells

The shapes of cells are the result of a balance between various physical forces operating on the cell envelope (Huang et al., 2012). These include a force akin to surface tension which operates on the cell’s lipid membrane and tends to minimise surface area, promoting a spherical shape; hydrostatic pressure from the cytoplasm (also known as ’turgor’ pressure), which depends on the difference in concentration of solutes between the cell and its surrounding medium (a balance which cells carefully regulate in various ways); and various contractile forces generated by the actin filaments within the cell (Harold, 2001, p. 127). In addition, bacteria and plant cells are surrounded by a rigid cell wall (made of peptidoglycan and polysaccharides like cellulose, respectively), which balances hydrostatic pressure and maintains cell shape. In cells without a rigid cell wall, like animal cells, the cytoskeleton is mainly responsible for maintaining cell shape, but mechanical forces external to the cell, including interactions with adjacent cells and with the extracellular matrix, also partly determine the shapes of cells. Both *in vivo* and *in vitro*, animal cells assume distinct shapes depending on the substrate on which they are growing, and even respond differently to the same molecular growth factors (Gospodarowicz et al., 1978).

The shapes of cells are related to their functionality, both in unicellular and multicellular organisms. For example, the only bacteria that are able to achieve very large sizes are extremely long and filamentous (e.g., Volland et al., 2022). This is due to the fact that bacteria experience severe energetic constraints when increasing their size because, for cells with similar shape, volume always increases more steeply than surface area, and metabolic demands usually scale with volume, whereas metabolic capacity in bacteria (which lack mitochondria) scales with surface area.

One of the main advantages of multicellularity is that it makes division of labour among cells possible, and different types of cells assume different shapes which are related to their functionality. For instance, red blood cells, especially in mammals, are extremely specialised and even lose their nucleus during differentiation. Their unique biconcave shape increases surface area and, because they are actually slightly larger than the diameter of capillaries, enables deformability which allows them to pass through (Harvey, 1997). This tight fit in turn forces the plasma between successive cells to flow faster than it would otherwise, thus increasing exchange between plasma and capillary walls (Vogel, 2025, p. 129). Epithelial, muscle, and neuron cells are also highly specialised for their distinct functions, which is reflected in their distinctive shapes. But even within each of these kinds of cells, there is remarkable diversity in structure and shape, which we are only beginning to understand. Recent studies on neuronal types indicate far more morphological variation than previously recognised, and in ways that reflect their synaptic connections (Masland, 2004). This morphological diversity is a significant source of complexity of the nervous system which is not easily accounted for by neuroscience models that consider the neurons as interchangeable nodes in a network.

### The shapes of organisms and their parts

As with molecules and cells, the shape of multicellular organisms and their macroscopic parts is also linked to their functionality in various ways. The whole body, as well as more specific parts of organisms such as limbs, teeth, feathers, etc., have been shaped by selective pressures for improved performance on activities that are relevant for organismal survival, such as food acquisition and locomotion, which involve specific kinds of physical interactions with the environment (understood to include both biotic and abiotic components).

For example, flying is a highly efficient but very energetically demanding mode of locomotion. Besides the downward pull of gravity, which the animal must counter through structures that provide lift (such as wings), flying organisms also experience drag, a physical force that tends to decelerate them, as they move through the air. Countering drag requires more energy to produce more thrust (for example, by increasing wing flapping frequency). Reducing drag allows an animal to achieve higher speeds and/or fly longer distances while spending the same energy or, conversely, to reduce energy requirements for the same performance, so most flying animals are under selective pressure to reduce drag (Alexander, 2015).

For large flying animals, streamlining and smoothing the body surface significantly reduce drag, so these modifications tend to be favoured by natural selection, resulting in the typical streamlined, aerodynamic shapes of birds. In contrast, for small flying organisms like most insects, streamlining is much less effective, because at low Reynolds numbers, air viscosity becomes more important; for these animals, becoming more spherical to reduce surface area is more effective (Alexander, 2015, p. 29). Thus, the shapes of organisms tend to evolve in ways that maximize performance in activities relevant for their survival, when interacting with the prevailing physical forces at their scale.

## A sensible reductionism

Spatial structure plays an important role in the functioning of organisms, cells, subcellular components, and biological systems in general. Neglect of this aspect of biological organisation is one of the limitations of genetic or molecular reductionism, which is rightly criticised for this reason. Recognition of this does not motivate organicism. On the contrary, spatial structure is a *physical* aspect of living systems and their parts, and the ways in which it makes a difference to various biological processes can often be understood from a physical perspective. While anti-reductionists are right to point out that it is not possible to explain the functioning of biological systems in terms of their components behaving as they do in isolation, this is also so true of non-living complex systems. Sensible reductionism does not preclude an explanation of the functioning of highly complex biological systems in terms of their components, their spatial relations, and their interactions, including interactions with things located outside of the system.

There is a long history of criticisms of reductionism appealing to spatial structure. However, argued above, spatial structure is essential to physics – for example, the shape of an object affects how it moves in fluids; as well as chemistry – for example, chemical isomerism. Therefore, spatial structure is essential to physical reduction. Frost-Arnold notes how “the appeal to the significance of gross spatial organization is a classical tenet of anti-reductionists in the biological sciences”, and worries that, “if we allow the reductionist to co-opt tenets that partly define anti-reductionism, then the distinction between the two camps disappears” (Frost-Arnold, 2004, p. 86). But the worry is misplaced, because there is a crucial difference between the two camps: where organicists understand biological organisation as a fundamental biological concept that cannot be explained in terms of anything else (Mossio, 2024, p. 9), something that marks “a dividing line between biological and physicochemical entities” (Sartenaer, 2024, p. 104), physical reductionists understand it in terms of the relative positions of objects in space, together with their physical interactions.

The complexity of living systems is a reason in favour, rather than against, *sensible* reductive approaches. To understand life fully we must understand how life emerges from physical and chemical processes. This cannot be done by taking the highly complex organisation of biological systems such as cells and organisms as inexplicable in terms of anything else. On the contrary, we can make progress in understanding life by explaining complex organisation in terms of spatial structure.

## Footnotes

1 Agency and autonomy are not discussed in this paper and it is not argued here that they can be reduced. There are many different forms of antireductionism, emergentism, holism, and organicism; our critique is focused on the denial of physical reduction and organicism in so far as it denies physical reduction.

2 We also note that there are contexts in which physical reduction is inadequate. These include, for example, functional explanations in evolutionary biology, and the requirement for evolutionary or ultimate causation in a complete explanation of an organism’s traits (Bock, 2017; Mayr, 1961). See Hermida and Ladyman (2025, 5) for further discussion.

3 Note that mereology – the study of parts and wholes – neglects interactions and so is different from scientific part-whole reduction.

4 For a fuller analysis of the relationship between physics and biology, and how physics both constrains and enables biological functioning, see Hermida and Ladyman (2025, 5).

5 Genetic reductionism as characterised here does not entail that human behaviour is controlled by genes (genetic determinism).

6 See Niklas et al. (2026﻿) for discussion of this example.

7 Although not all definitions of life take metabolism to be its most important feature, even those that prioritise other features, such as Darwinian evolution, also assume the existence of a population of metabolic entities. For example, NASA’s definition of life as “a self-sustaining chemical system capable of Darwinian evolution” clearly states that metabolism is included in the “self-sustaining” part of the definition (Joyce, 1994).

8 For an overview of the various roles that cell membranes and other physical boundaries play in living organisms, see Schulze-Makuch and Irwin (2018) and Hermida and Ladyman (2022)

9 Viruses are not a putative exception to the rule. While most biologists do not consider viruses to be living organisms, those that do appeal to the existence of an entity, the *virocell*, i.e., the infected cell where both metabolic activity and viral replication take place (e.g., Forterre, 2016), and this cell does of course have a membrane.

10 Regeneration of the cell membrane is essential. Sub-protoplasts that fail to regenerate a cell membrane degenerate (Kim et al., 2001, p. 2011).

11 A related, but different, notion of ‘boundary’ seems to be at stake in claims about the “looseness of boundary” between different organisms in symbiotic associations (Dupré, 2025, p. 45), which relates to the difficulty of distinguishing between biological systems involved in the same metabolic processes, such as holobionts. We have no objection to this claim. Note that the existence of a *physical* boundary between living beings and their environments is perfectly compatible with, for example, endosymbionts living *inside* the larger organism’s boundary, across which metabolic exchanges take place. We are grateful to an anonymous reviewer for raising this issue.

12 The same happens with mitochondria and plastids, which retain ribosomal DNA of their own and make their own ribosomes where they synthesize their own proteins.

13 This information can correspond to a sequence of RNA which, if translated, corresponds to a sequence of amino acids in a protein; or it can indicate when to start and when to stop reading; or it can be a regulatory factor; etc.

14 Bellazzi (2026) considers protein shapes as the bearers of functions for proteins.
